# *Dgcr8* and *Dicer* are essential for sex chromosome integrity during meiosis in males

**DOI:** 10.1242/jcs.167148

**Published:** 2015-06-15

**Authors:** Andrew J. Modzelewski, Stephanie Hilz, Elizabeth A. Crate, Caterina T. H. Schweidenback, Elizabeth A. Fogarty, Jennifer K. Grenier, Raimundo Freire, Paula E. Cohen, Andrew Grimson

**Affiliations:** 1 Departments of Biomedical Sciences, Cornell University, Ithaca, NY 14853, USA; 2 Molecular Biology and Genetics, Cornell University, Ithaca, NY 14853, USA; 3 Unidad de Investigacion, Hospital Universitario de Canarias, Ofra s/n, La Cuesta, La Laguna, Tenerife 38320, Spain

**Keywords:** *Dgcr8*, *Dicer*, *Dicer1*, Germ line, MicroRNA, Meiosis, Sex chromosomes, Sex body, Telomere

## Abstract

Small RNAs play crucial roles in regulating gene expression during mammalian meiosis. To investigate the function of microRNAs (miRNAs) and small interfering RNAs (siRNAs) during meiosis in males, we generated germ-cell-specific conditional deletions of *Dgcr8* and *Dicer* in mice. Analysis of spermatocytes from both conditional knockout lines revealed that there were frequent chromosomal fusions during meiosis, always involving one or both sex chromosomes. RNA sequencing indicates upregulation of *Atm* in spermatocytes from miRNA-deficient mice, and immunofluorescence imaging demonstrates an increased abundance of activated ATM kinase and mislocalization of phosphorylated MDC1, an ATM phosphorylation substrate. The *Atm* 3′UTR contains many potential microRNA target sites, and, notably, target sites for several miRNAs depleted in both conditional knockout mice were highly effective at promoting repression. RNF8, a telomere-associated protein whose localization is controlled by the MDC1–ATM kinase cascade, normally associates with the sex chromosomes during pachytene, but in both conditional knockouts redistributed to the autosomes. Taken together, these results suggest that *Atm* dysregulation in microRNA-deficient germ lines contributes to the redistribution of proteins involved in chromosomal stability from the sex chromosomes to the autosomes, resulting in sex chromosome fusions during meiotic prophase I.

## Abstract (precis)

**Highlighted Article:** miRNA-deficient spermatocytes display frequent sex chromosome fusions and fail to progress through meiosis in a process that is probably mediated by dysregulation of *Atm*.

## INTRODUCTION

Small RNA-mediated silencing has emerged as a major mechanism of gene regulation in animals and plants, with clear evidence for both cytoplasmic and nuclear small-RNA-based regulatory pathways that act upon RNA and DNA targets (Fire et al., 1998; Ketting, 2011; Matzke and Birchler, 2005; Wassenegger et al., 1994). Since the discovery of microRNAs (miRNAs) and endogenous small interfering RNAs (siRNAs), comparatively few studies have investigated roles for these small RNA species in mammalian reproduction. In females, siRNAs (but not miRNAs) are essential during oogenesis for proper maturation, meiotic spindle organization and chromosome alignment (García-López and del Mazo, 2012; Ma et al., 2010; Suh et al., 2010). During male gametogenesis, a third class of small RNAs, the piwi-interacting RNAs (piRNAs), are expressed in addition to miRNAs and siRNAs. The functions of piRNAs in the male germ line are relatively well-defined: piRNAs deter transposon integration, and also have distinct functions during prophase I (Yadav and Kotaja, 2014). In contrast to piRNAs, the roles and importance of miRNAs and siRNAs in the male germ line are poorly defined. Although it is clear that a functional miRNA pathway is necessary to complete spermatogenesis (Hayashi et al., 2008; He et al., 2009; Maatouk et al., 2008; Papaioannou and Nef, 2010; Zimmermann et al., 2014), the identities of essential miRNAs, and their mRNA targets, remain to be determined. Whether siRNAs are also required in the male germ line is presently unclear and remains controversial, with reports suggesting either that they have no role in spermatogenesis (Hayashi et al., 2008) or implicating siRNAs in specific functions in the germ line (Wu et al., 2012).

The major mode of action for miRNAs, based on a number of studies, is to accelerate the turnover of target mRNAs and inhibit their translation (Ketting, 2011; Meister, 2013). Such activities are consistent with the typical cytoplasmic localization of Argonaute (AGO) proteins, the RNA-binding proteins that function in concert with small RNAs and underpin all small RNA regulatory pathways (Cenik and Zamore, 2011; Meister, 2013). Additionally, in non-mammalian organisms such as nematodes, fungi and plants, Argonaute proteins have been shown to regulate chromatin structure and transcriptional activity (Maine, 2010; Shiu et al., 2001). Similar nuclear functions have yet to be firmly established in mammals, although some studies have hinted at their existence (Meister et al., 2004; Robb et al., 2005). Importantly, although mammalian AGO proteins have been observed in the nucleus, the functional significance of this localization is currently the subject of some debate (Chu et al., 2010; Tan et al., 2009).

We previously described the nuclear localization of AGO4, one of four mammalian Argonaute proteins, in mammalian germ cells during prophase I of meiosis. AGO4 accumulates within the sex body of pachytene spermatocytes. The sex body is a specialized nuclear subdomain harboring the X and Y chromosomes, in which the chromatin is transcriptionally silenced: a process known as meiotic sex chromosome inactivation (MSCI). In addition, AGO4 localizes to autosomal regions that fail to pair with their homologous partners; these unpaired regions are also silenced during prophase I as part of the more global meiotic silencing of unpaired chromatin (MSUC). *Ago4^−/−^* male mice are subfertile, with reduced testis size and lowered epididymal sperm counts. Meiotic prophase I progression is severely impaired in the absence of AGO4, resulting in a high proportion of spermatocytes undergoing apoptosis. Importantly, the formation and function of the sex body during pachytene of prophase I requires AGO4, given that MSCI is perturbed in *Ago4^−/−^* males (Modzelewski et al., 2012). Collectively, these data suggest that there is a direct role for AGO4 in transcriptional silencing during mammalian meiosis, specifically in regions of the genome in which chromosomes are unpaired (such as the X and Y chromosomes or asynapsed regions of the autosomes), in a fashion reminiscent of analogous processes in *Caenorhabditis elegans* and *Neurospora crassa* (reviewed by Maine, 2010).

Two lines of evidence suggest that AGO4 is not the sole Argonaute protein contributing to MSCI. First, AGO3 expression is upregulated in the absence of AGO4, and this upregulation is specific to the male germ line and specific to AGO3 alone (Modzelewski et al., 2012). Second, like AGO4, AGO3 localizes to the sex body during prophase I, suggesting that AGO3 and AGO4 function redundantly in mammalian meiosis. These observations imply that our analysis of *Ago4* mutant animals provides only a glimpse of the repertoire of siRNA and miRNA actions during meiotic prophase I.

In the current study, we created germline-specific conditional deletions of two proteins needed for small RNA biogenesis, DGCR8 and DICER (also known as DICER1). DGCR8, a component of the endonuclease microprocessor complex, is essential for the first step in miRNA processing – conversion of the primary miRNA transcript into a precursor miRNA. DICER, a second endonuclease, acts on the precursor miRNA in the cytoplasm to generate small RNAs, and is needed for the synthesis of almost all miRNAs (Kim et al., 2009; Murchison and Hannon, 2004). DICER, but not DGCR8, is also required for the processing of siRNAs (Bartel, 2004; Denli et al., 2004; Gregory et al., 2004; Han et al., 2004). Phenotypes common to both *Dgcr8* and *Dicer* conditional deletion animals would reveal roles of miRNAs in mammalian meiotic progression, whereas phenotypes specific to *Dicer* conditional deletion animals would likely implicate siRNAs. Our results demonstrate that loss of miRNAs leads to misregulation of the DNA damage repair pathway, including upregulation of *Atm* and improper localization of ATM substrate proteins. We find that the majority of these miRNA-deficient spermatocytes display frequent sex chromosome fusions and fail to progress through meiosis.

## RESULTS

### Conditional deletion of *Dgcr8* or *Dicer* in the male germ line results in sterility

To investigate and compare roles for miRNAs and siRNAs in the male germ line, we generated germline-specific deletions of *Dgcr8* and *Dicer*, which we analyzed in parallel. Our strategy used floxed alleles of *Dgcr8* and *Dicer*, in combination with a *Ddx4*-promoter-driven *Cre* transgene. The *Ddx4-Cre* transgene induces expression of the recombinase in spermatogonia from embryonic (e) day 18, before the initiation of meiosis at around day 8 post-partum (pp), but after the male-specific block of meiotic entry at around e12 (Gallardo et al., 2007). We selected this *Cre* transgene to allow unaltered development of both pre-meiotic germ cells and somatic compartments of the testis, while ensuring that all spermatocytes possess *Dgcr8* and *Dicer* deletions prior to meiotic initiation.

**Fig. 1.**
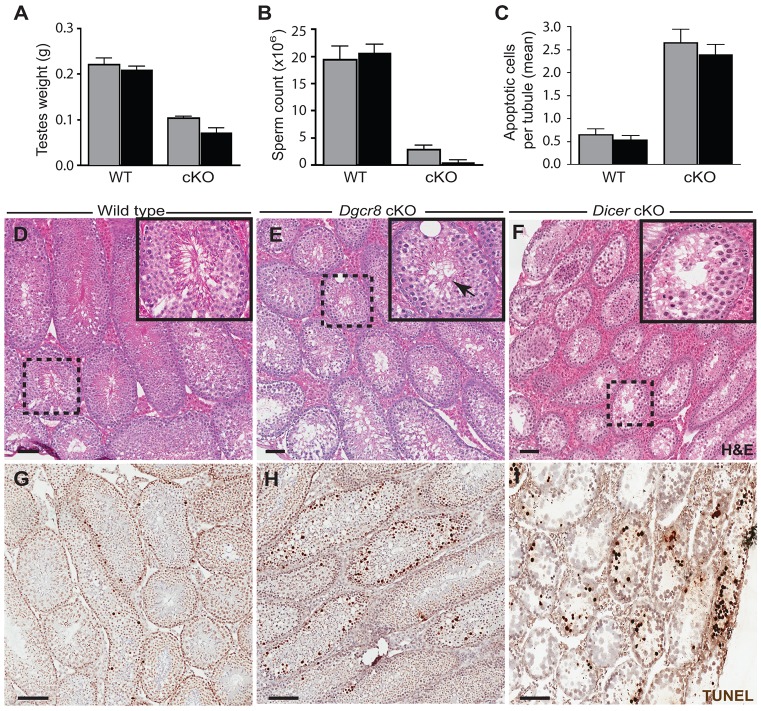
**Phenotypic analysis of *Dgcr8* and *Dicer* cKO males.** (A) Testis weights from *Dgcr8* cKO mice (gray bars; mean, 0.10 g, *n*=4) and *Dicer* cKO mice (black bars; mean, 0.07 g, *n*=4) are reduced compared to wild-type (WT) littermates (mean, 0.22 g, *n*=4, *P*<0.0001, *t*-test, and, mean=0.21 g, *n*=5, *P*<0.0001 for WT littermates of *Dgcr8* and *Dicer* cKO litters, respectively). (B) Caudal epididymal spermatozoa counts were significantly lower in *Dgcr8* cKO animals (*P*=0.0007) than in WT littermates (gray bars). Epididymal spermatozoa were rarely detected in *Dicer* cKO animals (two of four mice; *P*<0.0001) when compared to WT littermates (black bars). (C) Quantification of TUNEL staining from testes sections. TUNEL-positive cells were counted from multiple tubules (number specified below) from three mice for each condition to determine the mean number of apoptotic cells per tubule. In both *Dgcr8* and *Dicer* (gray and black bars, respectively) cKO testes, there were significant increases in apoptotic cell counts compared to WT littermates (*Dgcr8*, WT *n*=152, cKO *n*=262; 4.07-fold increase, *P*<0.0001; *Dicer*, WT *n*=154 cKO *n*=299; 4.43-fold increase *P*<0.0001). Error bars indicate s.e.m. (D–F) Testes sections from WT (D), *Dgcr8* cKO (E) and *Dicer* cKO (F) mice stained with H&E. Vacuous tubules were observed in both sections of *Dgcr8* (∼50% of tubules, E, inset) and *Dicer* (∼50% of tubules, F, inset) cKOs, but never in sections from wild-type littermates (D). The black arrow indicates tubules producing fully elongated spermatozoa in *Dgcr8* cKO animals (E). (G–I) TUNEL staining of testis sections from wild-type (G), *Dgcr8* cKO (H) and *Dicer* cKO (I) mice, with brown precipitate indicating cells undergoing apoptosis. Scale bars: 100 µm.

Conditional knock-out mice (cKO) of either *Dgcr8* or *Dicer* are infertile, as no offspring were recovered from matings to wild-type (C57BL/6) females over a 7-day period, despite normal mating behavior. At day 70 pp, both cKO mice exhibited normal body size (supplementary material Fig. S1C), but testis weights were significantly reduced compared to wild-type littermates (Fig. 1A and supplementary material Fig. S1A,B). Mean epididymal spermatozoa counts from *Dgcr8* and *Dicer* cKOs were also greatly reduced (Fig. 1B), with the degree of reduction significantly more severe in *Dicer* cKO germ lines (*P*<0.05, Student's *t*-test). Histological examination of testes from cKO animals revealed severe morphological abnormalities throughout the seminiferous epithelium, including an increased prevalence of vacuous tubules, together with tubules in which spermatogonia were arrested prior to the onset of spermiogenesis (Fig. 1E,F, insets). No mature spermatozoa were observed in *Dicer* cKO males, whereas mature spermatozoa, although rare, were detectable in *Dgcr8* cKO sections (Fig. 1E, inset arrow). Consistent with the absence of mature spermatozoa, the frequency of apoptotic spermatocytes, as assessed by TUNEL labeling, was also markedly increased in both *Dgcr8* and *Dicer* cKO testes (Fig. 1C,H,I). Overall, these results are consistent with observations from the majority of published studies of *Drosha*, *Dgcr8* and *Dicer* germline cKO mutants (Greenlee et al., 2012; Hayashi et al., 2008; Korhonen et al., 2011; Maatouk et al., 2008; Romero et al., 2011; Wu et al., 2012; Zimmermann et al., 2014). Our data implicate small RNAs that rely on both *Dgcr8* and *Dicer* processing, such as miRNAs, rather than *Dgcr8*-independent small RNAs, such as siRNAs, as the major non-coding small RNA regulators during early spermatogenesis.

### Sex chromosomes fail to synapse and frequently undergo chromosomal fusion in *Dgcr8* and *Dicer* cKO germ lines

**Fig. 2.**
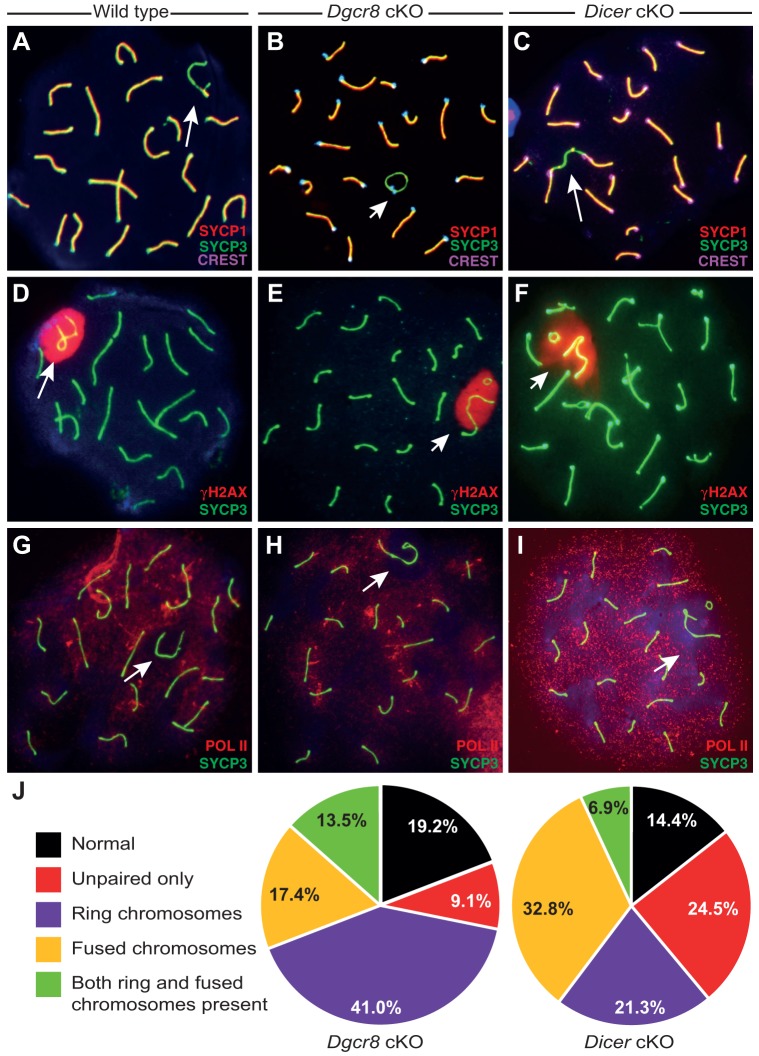
**Loss of DGCR8 or DICER results in chromosomal fusion abnormalities.** (A–I) Pachytene stage spermatocytes from wild-type (A,D,G) *Dgcr8* cKO (B,E,H) or *Dicer* cKO (C,F,I) mice, each stained with anti-SYCP3 antibody (green). XY chromosomes are indicated by white arrows. Immunostaining was also performed with antibodies specific to SYCP1 and CREST, a human autoimmune serum used to visualize centromeres (A–C, red and purple, respectively), γH2AX (D–F, red), and RNAP II (G–I, red). (J) *Dgcr8* cKO and *Dicer* cKO spermatocytes exhibit frequent chromosomal abnormalities. Chromosome spread preparations of pachytene stage spermatocytes were assessed for the types and frequencies of observed abnormalities, none of which were ever observed in wild-type pachytene spermatocytes. These were distributed into five indicated categories based on observed frequencies of X and Y chromosomal structures.

To investigate possible roles for small RNAs in spermatocyte nuclei, we examined prophase I chromosome spread preparations with antibodies raised against components of the synaptonemal complex, a meiosis-specific structure that connects homologous chromosomes during prophase I (Meuwissen et al., 1992; Yuan et al., 2000). The status of the synaptonemal complex serves as an indicator of the stages and progression of prophase I. We used antibodies against the chromosome axis protein SYCP3, which localizes along homologous chromosomes prior to synapsis, and SYCP1, a component of the synaptonemal complex central element, which serves to tether homologous chromosomes together from zygotene of prophase I onwards. Given that SYCP1 associates only with synapsed chromosomes, it is normally excluded from the unpaired X and Y chromosomes, except at a short region of homology capable of synapsis, referred to as the pseudoautosomal region (PAR; Fig. 2A, white arrow). Additionally, to investigate sex body integrity, we examined localization of H2AX, a histone variant whose phosphorylation (resulting in γH2AX) is essential for MSCI. During pachytene, H2AX localizes exclusively to the sex body of normal spermatocytes and to any autosomal sites of asynapsis.

Examination of *Dgcr8* and *Dicer* cKO spermatocytes in prophase I revealed that SYCP1 and γH2AX were mislocalized in a minority of cells (∼10%), in a pattern similar to that of *Ago4^−/−^* animals (Modzelewski et al., 2012). More strikingly, we observed a high frequency of structural abnormalities involving the X and Y chromosomes of cKO spermatocytes, with very few cells exhibiting normal X–Y associations or a normal PAR structure (Fig. 2). A range of abnormalities were observed, including circularization of the X and Y chromosomes (Fig. 2B,E,F; quantified in Fig. 2J), terminal fusion of either the X or Y chromosome to an autosome (Fig. 2C,E; quantified in Fig. 2J) and increased frequency of PAR asynapsis. Such defects were never observed in wild-type control littermates (Fig. 2A,D,G), nor in *Ago4^−/−^* animals (A.J.M., unpublished observations). Notably, chromosomal fusions always involved at least one sex chromosome, and were never observed between autosomes.

Despite frequent fusions between sex chromosomes and autosomes in both cKO germlines, staining patterns of γH2AX (Fig. 2E,F), the kinase ATR, and globally against ATR and ATM phosphorylation substrates are largely unaltered (supplementary material Fig. S1), retaining exclusive X and Y chromosome localization and wild-type intensity. In various translocation and asynapsis models, the chromosome that becomes associated with the X or Y is usually engulfed by the silencing machinery and thereby silenced (Heard and Turner, 2011). In both *Dgcr8* and *Dicer* cKO spermatocytes, however, there is no expansion of the γH2AX domain in response to these chromosomal abnormalities, and autosomes fused to sex chromosomes do not convert into a pseudo-sex-body-like state.

In wild-type spermatocytes, RNA polymerase II (RNAPII) localizes throughout the nucleus during pachytene, except where it is actively excluded, such as at centromeres and the sex body (Fig. 2G). In a small fraction of spermatocytes from *Dgcr8* (∼9%) and *Dicer* (∼5%) cKO animals, RNAPII was found aberrantly localized within the sex body domain. However, for many instances of the most extreme XY abnormalities, such as X-to-autosome fusions, both the *Dgcr8* and *Dicer* cKO pachytene spermatocytes still excluded RNAPII from the X chromosome portion of the fusion, while normal RNAPII staining was seen on the fused autosome (Fig. 2H,I). This differential localization of RNAPII presumably maintains, at least partially, the silenced status of the X and Y and the active status of the fused autosome. To confirm this, we used quantitative real-time PCR (qRT-PCR) to quantify the transcript levels of *Zfy1* and *Zfy2* (Royo et al., 2010), key sex-chromosome-encoded genes that must be silenced for spermatocytes to exit pachytene. Neither transcript was consistently elevated during pachytene (supplementary material Fig. S1L). This observation is consistent with the ability of spermatocytes from *Dgcr8* cKOs to proceed to diplotene, despite displaying frequent sex chromosome fusions (supplementary material Fig. S1J,K).

### Altered distribution of CDK2 and RNF8 in *Dgcr8* and *Dicer* cKO spermatocytes

The frequent chromosomal fusions observed in prophase I spermatocytes of both *Dgcr8* and *Dicer* cKO germ lines implies that one or more miRNAs regulate crucial aspects of chromosomal integrity during meiosis. Moreover, the restriction of these fusion events to the sex chromosomes suggests that understanding the molecular basis for this phenomenon might reveal sex-chromosome-specific regulatory features unique to meiotic prophase I. Thus, we pursued two complementary strategies to characterize the chromosomal phenotypes observed in *Dgcr8* and *Dicer* cKO germ lines, and ultimately, to identify the specific small RNAs whose absence results in chromosomal fusions. Firstly, we assessed the chromosomal localization of proteins that might be involved in the fusion events, comparing their localization patterns in wild-type and cKO germ cells. We tested proteins involved in various aspects of meiosis, chromatin organization, telomere stability and non-homologous end joining. Secondly, we performed RNAseq on purified meiotic cells from wild-type and cKO germ lines and examined transcriptome profiles for signatures that might explain the underlying phenotype.

**Fig. 3.**
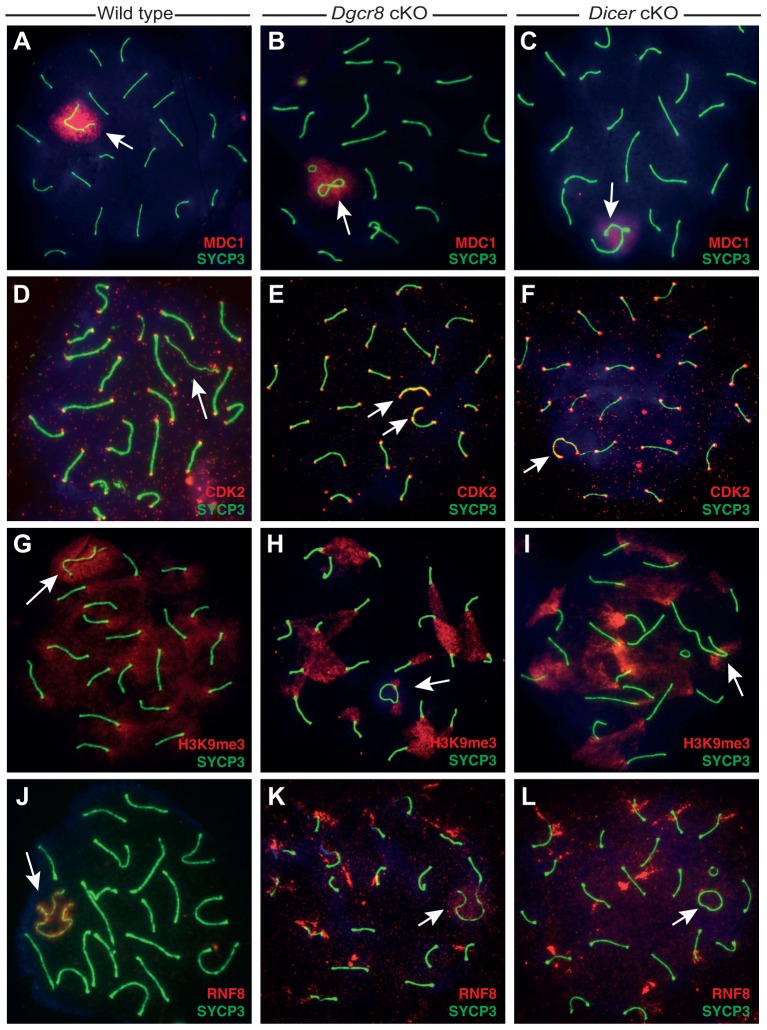
**Loss of DGCR8 or DICER disrupts localization of proteins associated with telomere maintenance.** (A–L) Pachytene stage spermatocytes from wild-type (A,D,G,J), *Dgcr8* cKO (B,E,H,K) or *Dicer* cKO (C,F,I,L) mice, each stained with anti-SYCP3 antibody (green). XY chromosomes are indicated by white arrows. Immunostaining was also performed with antibodies specific to MDC1 (A–C, red), CDK2 (D–F, red), H3K9me3 (G–I, red) and RNF8 (J–L, red).

Mediator of DNA damage checkpoint-1 (MDC1) is essential for spreading of DNA damage response factors after recognition of asynapsed and damaged chromatin. In wild-type pachytene spermatocytes, MDC1 localization is restricted to the sex body, similar to that of γH2AX (Fig. 3A). Almost all *Dgcr8* and *Dicer* cKO spermatocytes displayed normal MDC1 accumulation at pachytene, even in the presence of XY chromosomal abnormalities (Fig. 3B,C).

Cyclin-dependent kinase 2 (CDK2) plays multiple roles in spermatocytes. Loss of *Cdk2* results in infertility, a dramatic increase in synapsis between non-homologous chromosomes, the appearance of fusions and ring chromosomes, and eventual arrest in pachytene due to improper telomere maintenance (Parra et al., 2003). In wild-type pachytene spermatocytes, CDK2 localizes both to telomeres and sites of double-strand break (DSB) repair (Ashley et al., 2001) but is barely detectable on the unpaired XY chromosome core regions (Fig. 3D). In *Dgcr8* and *Dicer* cKO spermatocytes, however, the unpaired or circularized X and Y chromosome cores often (∼80% of spermatocytes in the *Dgcr8* cKO) displayed intense CDK2 localization across their entire length, that was not restricted to the telomeres (Fig. 3E,F).

Trimethylation of lysine 9 on histone 3 (H3K9me3) is an epigenetic mark associated with repressed heterochromatin. H3K9me3 is enriched at autosomal centromeres and the majority of the sex body in wild-type pachytene spermatocytes (Fig. 3G) (Kim et al., 2007; Tachibana et al., 2007). In a minor subset of cKO spermatocytes, we observed mislocalization of H3K9me3 throughout the meiotic nuclei; however, the majority of spermatocytes displayed unaltered localization of H3K9me3 despite exhibiting severe XY chromosomal abnormalities at pachytene (Fig. 3H,I).

The ubiquitin E3 ligase RNF8 ubiquitylates histones in response to DNA damage; RNF8 localization is controlled, in part, by interactions with phosphorylated MDC1 (pMDC1) (Mailand et al., 2007). Defects in RNF8 have been associated with chromosomal instability in cell culture – mouse embryonic fibroblasts (MEFs) lacking RNF8 show an increase in chromosomal aberrations, including ring chromosome formation (Rai et al., 2011). Normally, RNF8 localizes to the sex body in pachytene (Fig. 3J). However, in spermatocytes from *Dgcr8* and *Dicer* cKO males, RNF8 displays reduced sex body localization, instead redistributing along autosomes to flare-shaped domains that extend perpendicularly from the chromosome axes into the chromatin (Fig. 3K,L). In *Dgcr8* cKOs, we observed that RNF8 mislocalizes in 88% of spermatocytes with chromosomal abnormalities. These observations suggest that proper RNF8 localization is dependent, either directly or indirectly, on normal miRNA biogenesis. It is worth noting that in contrast to the rare aberrant localization of MDC1 and H3K9me3, the unusual localization of CDK2 and RNF8 in cKO germ lines was observed in the majority of abnormal pachytene spermatocytes.

### *Atm* is upregulated in *Dgcr8* and *Dicer* cKO spermatocytes

**Fig. 4.**
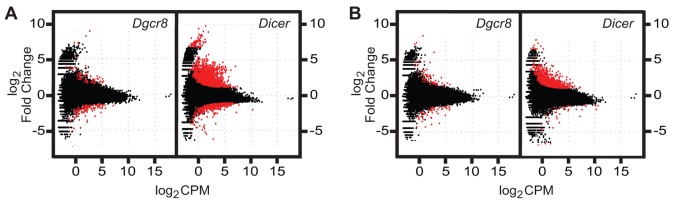
**Differential expression of genes in *Dgcr8* and *Dicer* cKO mixed leptotene and zygotene, and pachytene stage spermatocytes.** (A,B) The transcriptome was sequenced from purified mixed leptotene and zygotene (A) and pachytene (B) cells isolated from either *Dgcr8* or *Dicer* cKO mice, as well as from wild-type littermates as a control. Cells were derived from a minimum of two mice per genotype. Each individual cKO sample was compared to the corresponding wild-type littermate using the transcriptome sequencing analysis package edgeR to determine differential expression. Here, each transcript quantified is plotted by its average log_2_ CPM (counts per million, average of wild-type and cKO values) and the log_2_ of the fold change (cKO CPM/pooled wild-type CPM). In red are genes which are differentially expressed at a 5% false discovery rate (FDR). More genes were upregulated than downregulated in all samples (1.5-fold-change threshold), and more were differentially expressed in *Dicer* than in *Dgcr8* cKO samples (leptotene and zygotene, *P*<0.0001; pachytene, *P*<0.0001; χ^2^ tests).

To characterize dysregulation of mRNA expression levels in cKO spermatocytes, we profiled the transcriptome using RNAseq at pachytene and prior to pachytene (a mixed population of leptotene and zygotene spermatocytes). As parallel controls, we also profiled spermatocytes derived from wild-type littermates of both *Dgcr8* and *Dicer* breedings. Many transcripts exhibit significant differences in expression (Fig. 4A,B) between wild-type and cKO spermatocytes, with many more genes displaying such differences in *Dicer* cKO samples as compared to *Dgcr8* cKO samples (leptotene and zygotene, *P*<0.0001; pachytene, *P*<0.0001; χ^2^ tests). Our initial analyses of these data focused on miRNA targets, together with gene ontology analysis (supplementary material Fig. S3). As expected, the mRNAs upregulated in cKO samples were enriched (*P*<0.0001; χ^2^ test) in predicted miRNA target sites corresponding to miRNAs expressed in the germ line. However, a large proportion (26–31%) of genes upregulated in the cKO mutants do not contain predicted sites; such genes are likely downstream of regulatory events governed by direct targets, as are genes downregulated in the cKO mutants. The complexity of altered mRNA expression profiles is likely due to the multitude of dysregulated pathways resulting from loss of miRNAs. Candidate genes known to be involved in regulation of pathways important to telomere maintenance and chromosomal integrity were unchanged (supplementary material Tables S1 and S2). However, given the alterations in RFN8 localization and importance of DSB repair in telomere integrity, we noted that *Atm* is significantly upregulated in miRNA-deficient spermatocytes.

**Fig. 5.**
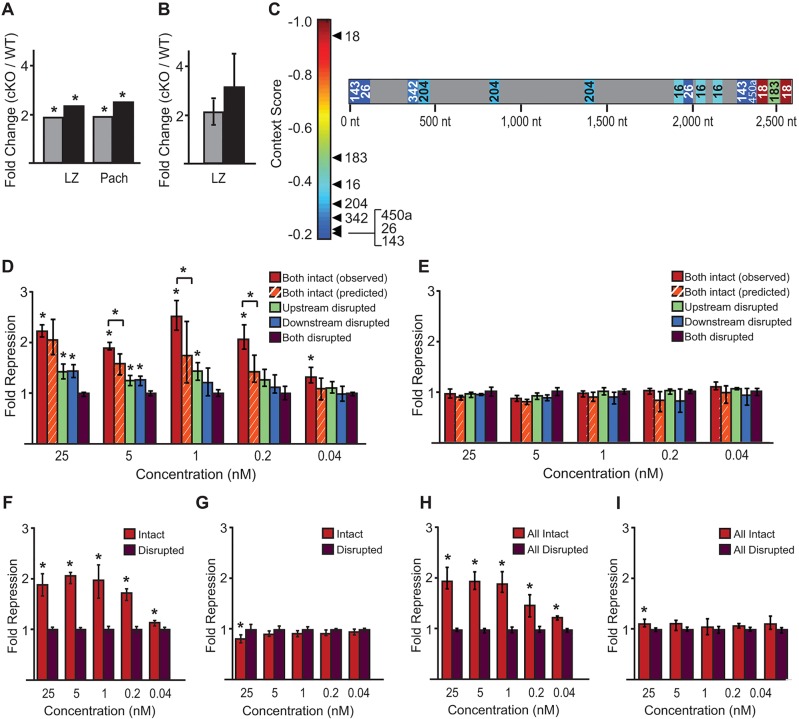
***Atm* expression is upregulated in *Dgcr8* and *Dicer* cKOs and a target of germline-expressed miR-18, as well as miR-183 and miR-16.** (A) Quantification of *Atm* by RNAseq of purified leptotene and zygotene (LZ) and pachytene (Pach) spermatocytes from *Dgcr8* (gray bars) and *Dicer* (black bars) cKO animals. The *y*-axis indicates fold change in read counts of each sample compared to wild-type samples. (B) qRT-PCR quantification of *Atm* from *Dgcr8* (grey bars) and *Dicer* (black bars) leptotene and zygotene spermatocytes plotted as fold change relative to wild-type transcript abundance. (C) Schematic of *Atm* 3′UTR (right) illustrating the position of all miRNA target sites corresponding to miRNAs expressed in the male germ line. Sites are color-coded by context score (Friedman et al., 2009; Garcia et al., 2011; Grimson et al., 2007; Lewis et al., 2005), which predicts site efficacy; increasingly negative scores indicate increasing predicted repression. For miRNAs with multiple target sites, the cumulative context score is plotted; only sites with a context score of −0.2 or below included. (D,E) Reporter assays of miR-18-mediated regulation of *Atm*. Luciferase reporter constructs containing a portion of the *Atm* 3′UTR (red bars) were transfected into A549 cells together with different concentrations (*x*-axis) of a miR-18 mimetic siRNA duplex (D) or miR-124, as a control (E). Fold regulation mediated by miR-18 was calculated by generating reporter constructs containing mutations disrupting both predicted target sites (purple bars) and normalizing luciferase activity to this construct. Similarly, to determine the efficacy of each individual site, reporter constructs containing mutations disrupting each individual site were also assayed (green and blue bars, representing disruption of the upstream and downstream site, respectively). *P<0.01 repression (*P*<7×10^−4^ for wild-type reporter at all concentrations, as well as reporters containing a single site at 25 and 5 nM; *P*<3×10^−3^ for the upstream site disrupted at 1 nM; Bonferroni-corrected Wilcoxon-rank sum tests). Orange-striped bars indicate the expected fold repression assuming that the two sites contribute independently to total repression (expected repression=repression of upstream site×repression of downstream site). Concentrations of miR-18 at which target sites contribute synergistically to repression are indicated with an asterisk and bracket above red and orange-striped bars; synergism was inferred when the observed repression of the *Atm* 3′UTR (red bar) significantly (*P*<0.01) exceeded that expected based on measurements of each site individually (orange-striped bar) (5 nM, *P*=0.0022; 1 nM, *P*=0.0029; 0.2 nM, *P*=0.0006; Bonferroni-corrected Wilcoxon-rank sum test). (F–I) Reporter assays of miR-183- and miR-16-mediated regulation of *Atm*, performed and analyzed as for miR-18. For miR-183, we compared the wild-type *Atm* construct (Intact, red bars) to one in which the single miR-183 site was disrupted (Disrupted, dark red bars) in the presence of a miR-183 mimetic (F) or a control miR-124 mimetic (G). For miR-16, we compared the wild-type *Atm* construct (All Intact, red bars) to one in which all three miR-16 sites are mutated (All Disrupted, dark red bars) in the presence of a miR-16 mimetic (H) or a control miR-124 mimetic (I).

**Fig. 6.**
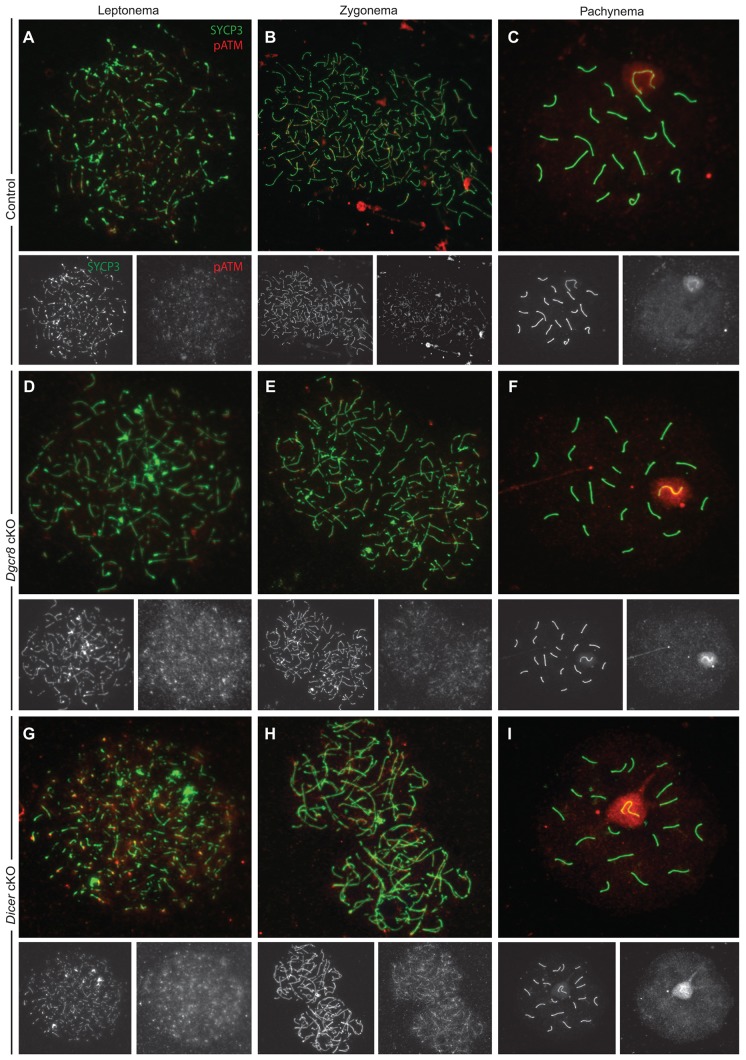
**Phosphorylated ATM protein localization is upregulated in prophase I chromosome spreads from *Dicer* and *Dgcr8* cKO males.** (A–I) Spermatocytes from wild-type (A–C), *Dgcr8* cKO (D–F) and *Dicer* cKO (G–I) were stained with anti-SYCP3 (green) and anti-pATM (S1981, red) antibodies. The red and green channels for each image are shown below the respective merged image. pATM levels are higher in the *Dicer* cKO (D,E) and *Dgcr8* cKO (G,H) as compared to wild-type (A,B) in leptotene and zygotene stage spermatocytes.

The kinase ATM plays multiple roles in DNA damage signaling and genome integrity, primarily acting through phosphorylation of MDC1. In response to phosphorylation, MDC1 recruits downstream targets including RNF8. The results of RNAseq on *Dgcr8* and *Dicer* cKO spermatocytes indicate that *Atm* transcript levels are increased, relative to wild-type, from leptotene through pachytene (Fig. 5A; supplementary material Table S3). We confirmed that *Atm* levels are upregulated in the mixed leptotene and zygotene samples from both *Dgcr8* and *Dicer* cKOs using qRT-PCR (Fig. 5B). Furthermore, immunofluorescence imaging of phosphorylated ATM (pATM), the activated form of ATM (Bakkenist and Kastan, 2003), demonstrates a subtle but discernible increase in protein levels during leptotene and zygotene (Fig. 6D,E,G,H). Moreover, transcriptionally regulated targets downstream of pATM are also upregulated (supplementary material Table S4). Thus, though other genes are also upregulated in *Dgcr8* and *Dicer* cKO spermatocytes, we focused on *Atm* owing to its known roles in promoting genomic stability and regulating RNF8 localization.

### MicroRNA-mediated regulation of *Atm* in the male germ line

The simplest explanation for elevated levels of *Atm* is that *Atm* is a target of germline miRNA(s), which are absent in both cKOs. To explore this possibility, we examined the *Atm* 3′UTR for predicted miRNA target sites, discovering many potential sites (Fig. 5C). To reduce the number of sites to those that would be targeted by co-expressed miRNAs, we sequenced small RNAs from the same samples used previously for RNAseq; this approach greatly reduced the number of sites worthy of scrutiny. Although predictions of target site efficacy are of limited accuracy (Thomas et al., 2010) we nevertheless focused on sites predicted by TargetScan (Friedman et al., 2009; Garcia et al., 2011; Grimson et al., 2007; Lewis et al., 2005) to be most effective in mediating repression. Strikingly, the miRNA predicted to mediate the strongest repression of *Atm* was miR-18, a miRNA known to exhibit meiosis-preferential expression (Björk et al., 2010). The target sites of two other miRNAs, miR-183 and miR-16, clustered in the same region of the *Atm* 3′UTR as the miR-18 target sites; moreover, the miR-183 and miR-16 target sites were predicted as the second and third strongest sites, respectively, within *Atm* (Fig. 5C). Furthermore, miR-18, miR-183 and miR-16 were among the most dramatically reduced miRNAs in *Dgcr8* and *Dicer* cKO spermatocytes, compared to wild-type (supplementary material Fig. S2J,M,N). Notably, none of the other miRNAs predicted to target *Atm* showed measurable depletion in the cKOs.

To investigate the efficacy of miR-18 target sites in the *Atm* 3′UTR, we designed a luciferase reporter construct containing 431 nucleotides of the endogenous *Atm* 3′UTR sequence encompassing the two predicted miR-18 target sites. We also generated otherwise identical reporters in which either one or both sites were disrupted. We measured reporter activity in a cell line that does not express miR-18 by co-transfecting the reporter plasmids with either an siRNA corresponding to miR-18 or a control siRNA corresponding to a miRNA that does not target *Atm*. Such reporter assays are typically performed with transfected siRNAs at a concentration of 20–100 nM, which results in a level of target repression comparable to that achieved by endogenous miRNAs expressed at high levels (Farh et al., 2005; Grimson et al., 2007; Krützfeldt et al., 2005; Rodriguez et al., 2007). Importantly, miR-18 is not one of the most abundant miRNAs in the murine male germ line (compare supplementary material Fig. S2J with supplementary material Fig. S2K,L). To measure the response of the *Atm* transcript to low levels of miR-18, we performed reporter assays using a range of concentrations of the miRNA mimetic, from 25 nM to 40 pM. We found that reporter constructs containing only a single miR-18 site were less effective at lower concentrations, whereas those with both sites intact elicit full repression over more than a 100-fold reduction in concentration of the miR-18 mimetic; repression at 0.2 nM was not significantly different from that measured at 25 nM (Fig. 5D). It is worth noting that the miR-18 target sites are located close to each other, an arrangement previously observed to mediate synergistic enhancements to repression by miRNAs (Grimson et al., 2007; Saetrom et al., 2007). To determine whether repression mediated by miR-18 sites in *Atm* is synergistic, we compared the observed repression mediated by both sites to a value extrapolated from that mediated by each individual site (Fig. 5D, red and orange-striped bars, respectively). Using this approach, we did not observe evidence of synergism at high concentrations (25 nM) of the miR-18 mimetic; at lower concentrations (5, 1 and 0.2 nM), however, we observed increasingly strong evidence for synergistic interactions between the sites (Fig. 5D). We repeated our reporter assay for the single miR-183 site, as well as for the three miR-16 sites. Like the miR-18 target sites, the single site for miR-183 (Fig. 5F) and combined miR-16 sites (Fig. 5H) were able to mediate an ∼2-fold repression of the reporter construct at high-to-moderate concentrations (25-nM–1-nM) of miR-183 and miR-16 mimetic. Notably, the level of repression (∼2-fold) corresponds well to the change in levels of the endogenous *Atm* transcript in cKO germ lines. Taken together, these results indicate that miR-18, miR-183, and miR-16 target sites in *Atm* are functional and effective at eliciting downregulation in response to very low levels of miRNA.

### Aberrant localization of pMDC1 to autosomes in *Dgcr8* and *Dicer* cKOs

**Fig. 7.**
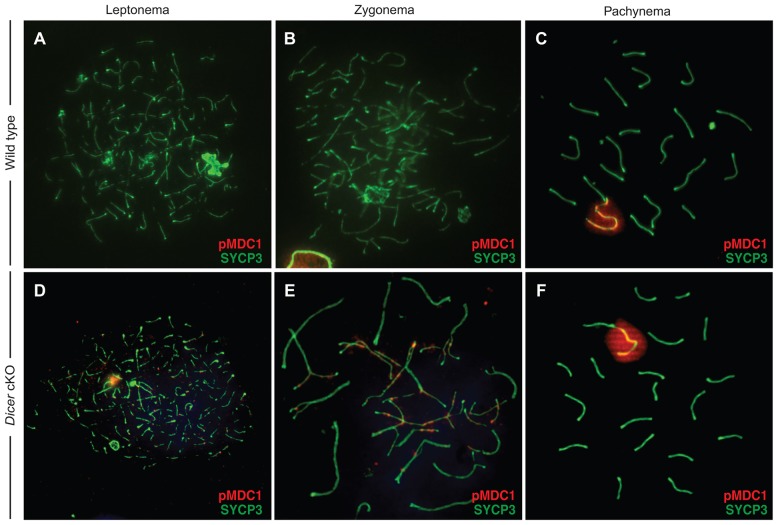
**Phosphorylated MDC1 mislocalization in *Dicer* cKO prophase I chromosome spreads.** (A–F) Spermatocytes from wild-type (A–C) and *Dicer* cKO (D–F) mice stained with anti-SYCP3 (green) and anti-pMDC1 (red) antibodies. pMDC1 was mislocalized in 80% of leptotene spermatocytes.

Our results implicate increased levels of ATM in driving RNF8 from the sex-body to the autosomes in miRNA-deficient spermatocytes. ATM does not directly recruit RNF8; rather, recruitment is mediated by ATM-catalyzed phosphorylation of MDC1 (pMDC1). Although MDC1 localization appears normal in the majority of *Dgcr8* and *Dicer* cKO spermatocytes at pachytene (Fig. 3B,C), we reasoned that any alteration in RNF8 localization at pachytene would more likely derive from altered pMDC1 distribution prior to this stage of meiosis, especially given the ∼2-fold increase in *Atm* expression seen in leptotene and zygotene. In wild-type pachytene spermatocytes, pMDC1 staining accumulates in the sex body (Fig. 7C), and localization of pMDC1 appears unaffected in *Dicer* cKO pachytene spermatocytes (Fig. 7F). At zygotene, however, pMDC1 is normally undetectable on the autosomes in wild-type spermatocytes (Fig. 7A,B, leptotene and zygotene, respectively), but is dramatically upregulated across the autosomes in *Dicer* cKO spermatocytes (Fig. 7D,E). Localization of the core MDC1 protein, regardless of its phosphorylation status, appears to be similar to the wild-type in both *Dgcr8* and *Dicer* cKOs (supplementary material Fig. S4), suggesting that the majority of the MDC1 pool remains unphosphorylated. These results, indicating elevated ATM kinase activity in *Dicer* cKO spermatocytes, confirm our observations of increased levels of the *Atm* transcript in *Dgcr8* and *Dicer* cKO germ lines. Taken together, our data suggest that loss of miRNA-mediated control of *Atm* initiates a series of events in cKO spermatocytes, titrating first pMDC1 and then RNF8 away from the sex body, and ultimately resulting in sex chromosomes that are deficient in their normal complement of DNA-damage surveillance and repair proteins.

## DISCUSSION

The roles and significance of miRNAs and siRNAs during meiotic progression in mammals are poorly understood. Here, we used mouse models to demonstrate that the loss of miRNAs in spermatocytes results in drastic alterations in sex chromosome morphology, typified by an increased rate of chromosome circularization and end-to-end fusions reminiscent of telomere fusions. These telomere-related events are associated with misregulation of the DNA damage repair pathway, including increased amounts of the *Atm* transcript*,* a greater abundance of ATM protein at leptotene and zygotene, and mislocalization of ATM substrates. We also identified alterations in many small RNAs, including miR-18, miR-183 and miR-16, among whose targets is the mRNA encoding ATM. Our results indicate that miRNAs play a critical role in regulating DNA damage repair machinery, and ultimately chromosome stability, during mammalian spermatogenesis.

Previous studies investigating conditional knock-outs of *Dgcr8*, *Drosha* and *Dicer* in the male germ line have noted many of the same gross morphological defects we observed, including infertility (or subfertility), decreased sperm count, and disrupted sperm morphology (Greenlee et al., 2012; Hayashi et al., 2008; Korhonen et al., 2011; Maatouk et al., 2008; Romero et al., 2011; Wu et al., 2012; Zimmermann et al., 2014). A variety of essential roles for small RNAs in spermatogenesis have been proposed, including silencing of expression from the X and Y (Greenlee et al., 2012; Wu et al., 2012), regulating SINE levels (Romero et al., 2011), repressing centromeric repeat transcripts (Korhonen et al., 2011) and functioning in DNA repair at DSBs (d'Adda di Fagagna, 2014). Our work provides an additional explanation for the requirement of small RNAs in the male germline – miRNAs play an essential role in spermatocyte development by directly regulating levels of the ATM kinase, resulting in relocalization of several DNA damage repair proteins and leading to chromosomal fusions. Although this study does not directly address whether the chromosomal fusions in *Dgcr8* and *Dicer* cKOs underlie the failure of such animals to complete spermatogenesis, such gross chromosomal abnormalities almost always lead to meiotic failure and failed chromosome segregation (Bolcun-Filas and Schimenti, 2012). Importantly, our model is based on the established biology of miRNAs (post-transcriptional regulation of mRNAs) and does not invoke new functions for miRNAs.

Many miRNAs are predicted to target the *Atm* 3′UTR, but only three are also expressed in spermatocytes and show depletion in *Dgcr8* and *Dicer* cKOs: miR-18, miR-183 and miR-16. We demonstrate that each of these miRNAs can effectively regulate the *Atm* 3′UTR, even at low concentrations; disruption of this regulation results in a ∼2-fold upregulation of reporter activity, roughly the same change we observe for *Atm* in miRNA-deficient spermatocytes. Therefore, miR-18, miR-183 and miR-16 are the strongest candidates for miRNA-mediated regulation of *Atm* expression in mammalian spermatogenesis.

Despite the observed upregulation of *Atm* in both mixed leptotene and zygotene, and pachtyene spermatocytes, the activated ATM substrate pMDC1 is only mislocalized during leptotene and zygotene. By pachytene, pMDC1 is restricted to the sex chromosomes in both cKO and wild-type mice. This result is congruent with previous studies that propose that there are two waves of phosphorylation during mammalian meiosis. The first wave is catalyzed by ATM during leptotene, in response to DSBs (Bellani et al., 2005). ATR kinase, a relative of ATM, is thought to initiate a second wave of phosphorylation during zygotene and pachytene. During zygotene, ATR localizes to sites of asynapsis on all chromosomes, and during pachytene it accumulates on the unpaired sex chromosomes, where it functions along with MDC1 and γH2Ax to silence the X and Y (Ichijima et al., 2011; Royo et al., 2013; Subramanian and Hochwagen, 2014). Therefore, normal localization of pMDC1 in pachytene is likely due to the ability of ATR to rescue MDC1 misregulation at this stage of meiosis. As MDC1 is essential for meiotic silencing, its normal localization on the sex body by pachytene explains why its earlier mislocalization does not result in a detectable defect in MSCI. We do see, however, that RNF8, which is recruited to sites of DSBs by pMDC1, persists on the autosomes during pachytene in *Dgcr8* and *Dicer* spermatocytes and appears depleted from the sex chromosomes. Taken together, these results suggest that although the activity of ATR at the sex body during pachytene is able to correct the mislocalization of some proteins that function downstream of ATM, the mislocalization of other downstream proteins persists throughout pachytene.

**Fig. 8.**
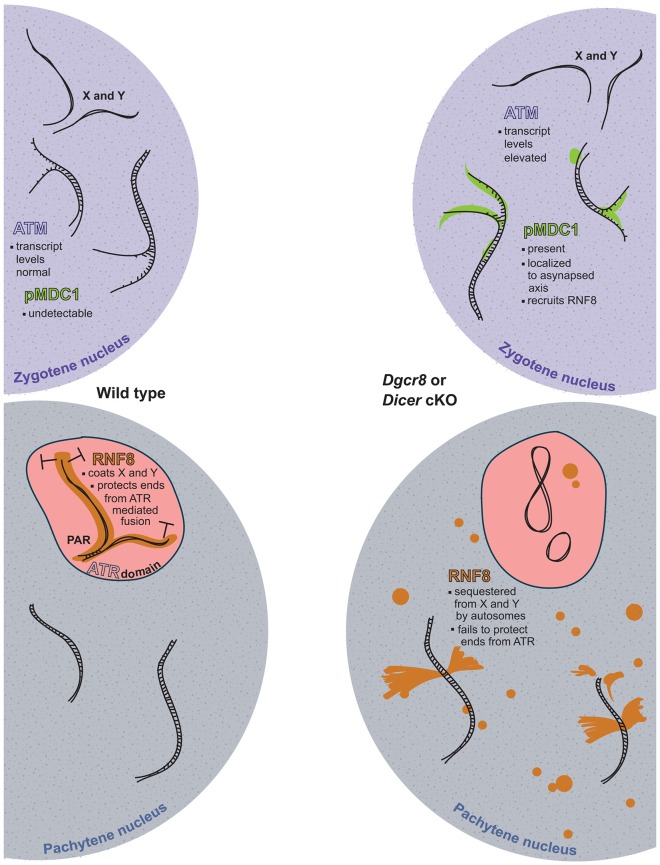
**Model for the role of miRNA-mediated regulation in controlling sex chromosome stability.** In wild-type males at the zygotene stage (upper left), pMDC1 is undetectable on both the autosomes and sex chromosomes. In *Dgcr8* and *Dicer* cKOs (upper right), pMDC1 is evident and localizes to asynapsed cores. In addition, the transcript encoding ATM, which phosphorylates MDC1, is upregulated at this stage in cKOs. By the subsequent pachytene stage, RNF8 (whose localization is driven by pMDC1) is enriched within the sex body in wild-type males (lower left), coating the X and Y chromosomes, where it functions to protect telomeres from triggering ATR. In males deficient for miRNAs (lower right), *Atm* elevation and the resulting pMDC1 mislocalization at zygotene recruits RNF8, and likely other members of the DNA damage repair pathway, to the autosomes. Sequestration of RNF8, and potentially other factors, away from the sex chromosomes deprotects telomeres, leading to ATR-mediated chromosomal fusions by NHEJ.

Although the *Dgcr8* and *Dicer* cKO spermatocytes exhibit misregulation of several proteins that have been previously implicated in chromosomal fusions, the precise contribution of these proteins to sex chromosome abnormalities is unclear. In particular, we observe misregulation of CDK2 and RNF8 in the *Dgcr8* and *Dicer* cKOs. *Cdk2*^−/−^ mice display frequent chromosomal fusions during meiosis, but importantly, these fusions never involve the X and Y (Viera et al., 2009). Furthermore, these defects are presumably due to an absence of CDK2 on chromosomes, and we see that CDK2 is present on the sex chromosomes in our cKOs. MEFs lacking RNF8 exhibit an increase in chromosome fusion events (Rai et al., 2011), but the role RNF8 plays in chromosome stability during meiosis is less clear. Mice lacking RNF8 are able to progress through meiosis with no observable chromosomal fusion, although post-meiotic defects result in abnormal spermatids and infertility (Lu et al., 2010). RNF8 has been implicated in playing opposing roles in fusion events at telomere ends, which can fuse through either classical (C-NHEJ) or alternative (A-NHEJ) non-homologous end joining (Rai et al., 2010). RNF8 is required for efficient telomere fusion through the ATM-dependent C-NHEJ pathway in mouse models that promote such fusions (Jacobs, 2012; Peuscher and Jacobs, 2011). However, RNF8 also protects telomere ends from undergoing fusion through the ATR-dependent A-NHEJ pathway by stabilizing the shelterin protein TPP1 (Rai et al., 2011). TRF1 (also known as TERF1), a member of the shelterin complex typically disrupted in C-NHEJ, appears intact in our cKO pachytene spermatocytes (supplementary material Fig. S4). Therefore, RNF8 redistribution from the sex body onto the autosomes in *Dgcr8* and *Dicer* cKOs might directly contribute to fusions involving the sex chromosomes by promoting an A-NHEJ-like mechanism (Fig. 8). Alternatively, we cannot exclude the possibility that mislocalization of RNF8 might be a downstream effect of other events occurring at the sex chromosomes that themselves lead to such fusion events.

An estimated 7% of the male population will encounter fertility problems, and in approximately half of these cases, the causes will be unknown (Krausz, 2011). Patients with Ataxia Telangiectasia, caused by *Atm* mutations, are often infertile, as are mice deficient in *Atm* (Barlow et al., 1996, 1998; Boder, 1975). However, whether *Atm* overexpression can lead to infertility is unknown; indeed, there is a notable absence of studies reporting the consequences of ATM overexpression. This study suggests that overexpression of *Atm* in spermatocytes, whether mediated by miRNAs or by other mechanisms, would likely cause fertility defects in the male germ line. Here, we demonstrated that miRNA-deficient spermatocytes display frequent chromosomal fusion events involving the sex chromosomes and upregulated *Atm* expression. Additionally, we observed mislocalization of other members of the DSB repair machinery downstream of ATM in *Dgcr8* and *Dicer* cKOs. Finally, we have identified candidate miRNA regulators of ATM expression in the male meiotic germ line. Our results underscore the significance of specific miRNAs in ensuring the fidelity of gametogenesis, and point to miR-18, miR-183 and miR-16 as miRNAs playing an important role in male fertility.

## MATERIALS AND METHODS

### Mouse breeding strategies

Female mice carrying homozygous floxed alleles for either *Dgcr8^Fl/Fl^* (C57BL/6 strain background, Rui Yi, Univeristy of Colorado, Boulder) or *Dicer^Fl/Fl^* (C57BL/6;129S7-Dicer1^tm1Smr^/J; Jax Stock 012284) were crossed to male mice carrying the *Ddx4*-cre transgene [FVB-Tg(Ddx4-cre)1Dcas/J; Jax Stock 006954] to generate the desired *Dgcr8^Fl/+^cre^+^* and *Dicer^Fl/+^* *cre*^+^ breeder males, which were then crossed to *Dgcr8^Fl/+^cre*^−^ and *Dicer^Fl/+^* *cre*^−^ breeder females, respectively. These crosses generated the experimental cohorts, which included male mice displaying the *Dgcr8^Fl/^***^Δ^***cre^+^* and *Dicer^Fl/^***^Δ^**cre^+^ genotypes, which were homozygous knockouts in the targeted germline cell types (referred to as conditional knockouts, cKO, *Dgcr8***^Δ^***^/^***^Δ^** *cre^+^* and *Dicer***^Δ/Δ^**cre^+^), together with wild-type (*+/+ cre^+^, +/+ cre^−^*, or *Fl/+ cre^−^*) littermates. Genotypes were confirmed using DNA isolated from tail snips and PCR assays specific to the wild-type, floxed and deleted alleles. All mice were fed *ad libitum* with standard laboratory rodent chow, and were maintained under controlled conditions of light and temperature, according to the regulations outlined and approved by the Cornell Institutional Care and Use Committee.

### Testes weights, sperm counts, histology and TUNEL staining

Whole testes were removed from wild-type and cKO littermates, weighed and epididymal sperm counts assessed (Edelmann et al., 1996). For histological analysis, testes were fixed in Bouin's fixative [hematoxylin and eosin (H&E) staining] or 10% formalin (TUNEL and all other staining) overnight at 4°C. Paraffin-embedded tissues were sectioned at 5 µm, and processed for H&E staining or immunohistochemical analyses using standard methods. TUNEL staining was performed using the Apoptag TUNEL staining kit (Chemicon, Temecula, CA).

### Chromosome spreading and immunofluorescence staining

Prophase I chromosome spreads, antibodies and antibody staining were as previously described (Holloway et al., 2008, 2011; Kolas et al., 2005; Lipkin et al., 2002; Modzelewski et al., 2012), except for antibodies recognizing ATR (GeneTex GTX70133), and ATR and ATM substrates (Cell Signaling #5851), RNA polymerase II (Millipore 05-623, Covance MMS-129R), MDC1 and RNF8 (Raimundo Freire, Tenerife, Spain), CDK2 (Abcam ab7954), H3K9me3 (Millipore 07-442), TRF1 (Abcam ab10579), ATM pS1981 (Rockland Antibodies 200-301-400), and pMDC1 (Abcam ab35967). Alexa-Fluor-conjugated secondary antibodies were used (Molecular Probes, Eugene, OR, USA) for immunofluorescence staining at 37°C for 1 h. Slides were washed and mounted with Prolong Gold antifade (Molecular Probes).

### Image acquisition

All slides were visualized using a Zeiss Imager Z1 microscope under 20×, 40× or 63× magnifying objectives, at room temperature. Images were processed using AxioVision (version 4.7, Zeiss).

### Statistical analysis

Statistical analyses (χ^2^ test, unpaired Student's *t*-test and Wilcoxon rank sum test) were performed using GraphPad Prism version 4.00 for Macintosh (GraphPad Software, San Diego, CA) and online web utilities (http://vassarstats.net and http://www.fon.hum.uva.nl/Service/CGI-Inline/HTML/Statistics.html).

### Isolation of mouse spermatogenic cells

Testes from adult *Dgcr8* and *Dicer* cKOs, together with wild-type littermates (day 70–80 pp, a minimum of two mice per genotype) were removed, weighed and decapsulated prior to enrichment of specific spermatogenic cell types using the STA-PUT method based on separation by cell diameter and density at unit gravity (Bellvé, 1993). The purity of resulting fractions was determined by microscopy based on cell diameter and morphology. Pachytene cells were ∼90% pure, with residual possible contamination from spermatocytes of slightly earlier or later developmental stages. Leptotene and zygotene cells were isolated in combination, and were >90% pure. Neither the pachytene nor the mixed leptotene and zygotene fraction visually exhibited evidence of contaminating somatic cells. RNA was extracted from STA-PUT purified cells using TRIzol (Life Technologies), and used as the source material for mRNA and small RNA sequencing and qRT-PCR.

### mRNA transcript sequencing and analysis

Non-stranded RNAseq libraries were prepared (TRUseq, Illumina) and sequenced (Illumina HiSeq 2500). The resulting sequences were mapped to the genome (mm9) using BWA (v0.7.8; Li and Durbin, 2009). The number of reads mapping to each transcript was quantified using HTSeq (v0.6.1; Anders et al., 2015), and differential transcript expression between samples was assessed using edgeR (v3.6.8; Robinson et al., 2010; R version: 3.1.0). Targeting analysis was performed using custom Python scripts and the TargetScan Mouse database (v6.2; Friedman et al., 2009; Garcia et al., 2011; Grimson et al., 2007; Lewis et al., 2005).

### Small RNA sequencing and analysis

Small RNA sequencing libraries were prepared (TruSeq Small RNAseq, Illumina) and sequenced on an Illumina HiSeq 2500 machine. High-quality reads were aligned to the genome (mm9) using Bowtie (v0.12.7; Langmead et al., 2009). Mouse miRNA hairpin sequences were obtained from miRBase (v20; Griffiths-Jones et al., 2006; Kozomara and Griffiths-Jones, 2014).

### Accession numbers

Deep sequencing files are available from NCBI GEO (GSE63166).

### qRT-PCR

Complementary DNA was synthesized with Transcriptor reverse transcriptase (Roche Applied Science) according to the manufacturer's instructions using 1 μg total RNA. qRT-PCR reactions were run in triplicate on a LightCycler480 (Roche Applied Science). A melt curve for each reaction confirmed amplicon identity, and a standard curve was used to calculate transcript abundance, assaying GAPDH (5′-TGAAGCAGGCA-TCTGAGGG-3′ and 5′-CGAAGGTGGAAGAGTGGGAG-3′); ATM (5′-TCAGGCTGTATCTCAAGCCAT-3′ and 5′-AAGGGCTGCTAAGATG-TGACT-3′).

### Luciferase assays

For the miR-18 assay, a 431 nt fragment of the *Atm* 3′UTR (chr9:53245275-53245705) containing the two miR-18 target sites was amplified from mouse DNA using primers 5′-TGAGTGAGACGGGCTGTTACC-3′ and 5′-TCCTGGACTGCCTACTGATTCC-3′, and reamplified using primers (cloning sites underlined) 5′-ATATGAGCTCTGCCTGAGGACAGAAG-ACATTG-3′ and 5′-ATATTCTAGATTCAGGAAACAGCATAACTGAA-AAAC-3′, digested and cloned into the luciferase reporter pmirGLO (Promega), to generate the miR-18 wild-type *Atm* reporter. For the miR-183 and miR-16 assay, a slightly larger fragment of the *Atm* 3′UTR, which spans 739 nt (chr9:53245260-53245998) was used so as to include all three miR-16 sites, in addition to the two miR-18 and one miR-183 sites. This fragment was amplified from mouse DNA using primers (cloning sites underlined) 5′-ATATGAGCTCTTCAGATTTCTTCAGTGGCTTTGATA-AATCTATGTC-3′ and 5′-ATATTCTAGAAAAACAAACTGATAATTC-A-GGAAACAGCATAACTG-3′, and cloned into pmirGLO (as described above), to generate the miR-183 and miR-16 wild-type *Atm* reporter. Reporter constructs in which one or more sites were mutated (QuikChange, Agilent) were generated using the wild-type reporter as a template. Sites were mutated as follows: the miR-18 site (GCACCUUA) was mutated to GCAggaUA for both miR-18 sites; the miR-183 site (GUGCCAUA) was mutated to GUGaCgUA, the miR-16 sites (GCUGCU) were mutated to either GCcGaU, GaUGgU, GCcGaU (corresponding to the order of the sites within the reporter construct). Reporter constructs were transfected into A549 cells along with varying levels of siRNA duplexes corresponding to miR-18 (using RNA oligonucleotides: 5′-UAAGGUGCAUCUAGUGCA-GAU-3′ and 5′-CUGCACUAGAUGCACCUUAAU-3′), miR-183 (5′-UA-UGGCACUGGUAGAAUUCACU-3′ and 5′-UGAAUUCUACCAGUG-CCAGAUA-3′), miR-16 (5′-UAGCAGCACGUAAAUAUUGGCG-3′ and 5′-CCAAUAUUUACGUGCUGUUAUU-3′), or, as a control, miR-124 (Lim et al., 2005). *Renilla* and firefly luciferase activity (Dual-luciferase assay, Promega) were measured (Veritas luminometer, Turner Biosystems) at 24 h post-transfection. To control for transfection efficiency, firefly luciferase values were normalized to those of *Renilla* luciferase. These values were then normalized to those of the construct in which all target sites for that particular miRNA were disrupted. Fold repression was calculated by taking the inverse of the fold change. Predicted fold repression for the wild-type reporter was calculated using measurements for each single mutant reporter (Grimson et al., 2007).

## Supplementary Material

Supplementary Material
