# Supplementary material for: Dgcr8 and Dicer are essential for sex chromosome integrity during meiosis in males
Source: J Cell Sci. 2015 Jun 15;128(12):2314–27. doi: 10.1242/jcs.167148 (PMC4487015; doi:10.1242/jcs.167148)
Supplement: Supplementary Material [file supp_128_12_2314__index.html]

Dgcr8 and Dicer are essential for sex chromosome integrity during meiosis in males — Supplementary Material 

# *Dgcr8* and *Dicer* are essential for sex chromosome integrity during meiosis in males

## JCS167148 Supplementary Material

**Files in this Data Supplement:**

- **Supplementary Material**
