# Supplementary material for: Dgcr8 and Dicer are essential for sex chromosome integrity during meiosis in males
Source: J Cell Sci. 2015 Jun 15;128(12):2314–27. doi: 10.1242/jcs.167148 (PMC4487015; doi:10.1242/jcs.167148)
Supplement: Supplementary Material [file supp_128.12.2314_JCS167148.pdf]

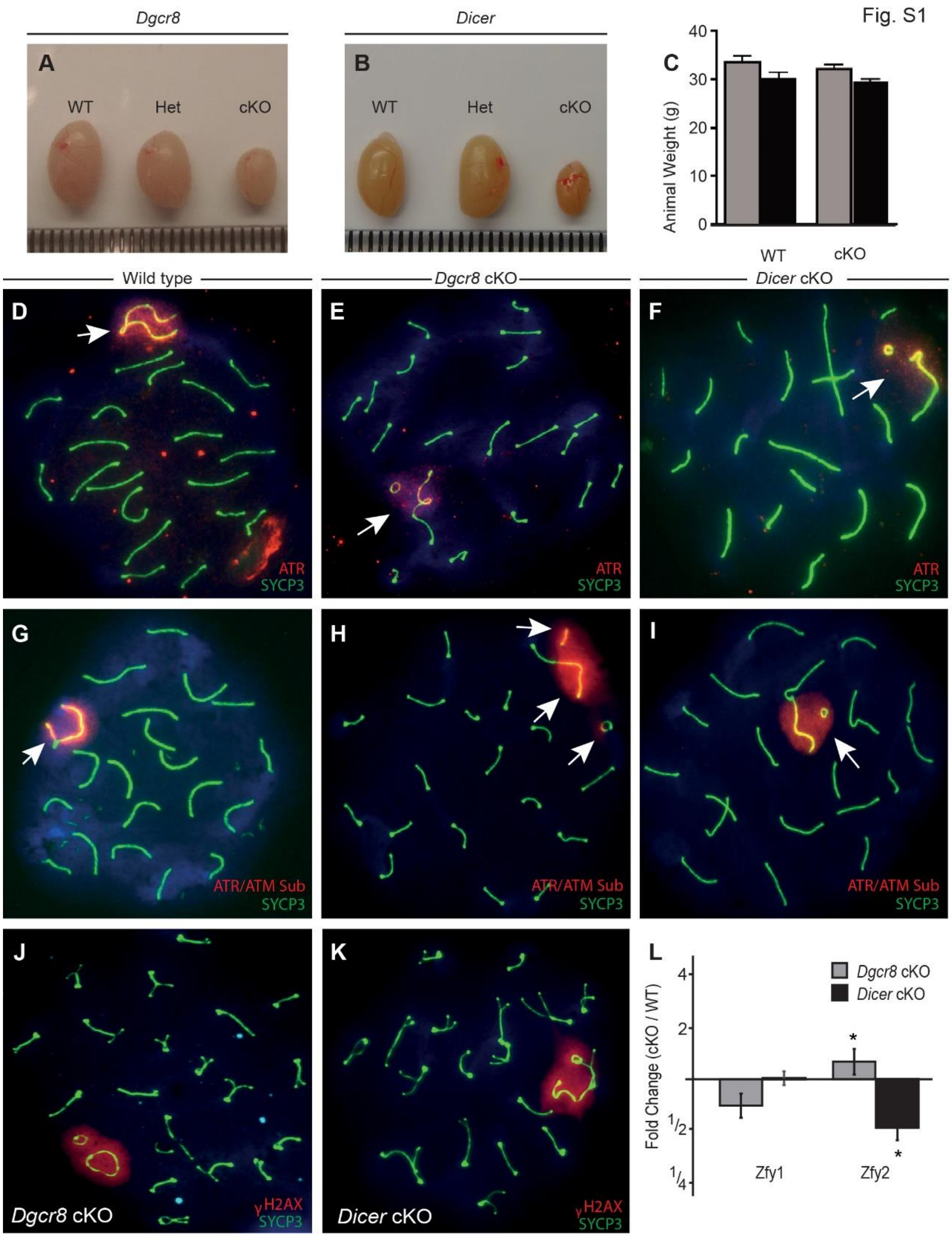

Fig. S1. Phenotypic analysis and assessment of meiotic silencing in *Dgcr8* and *Dicer* cKO males.

A-B Photographic representation of whole testes removed from *Dgcr8* (A) and *Dicer* (B) litters (wild type (WT): Fl/+, cre-, heterozygote (Het): Δ/+ cre and cKO: Fl/Δ cre+).

C Total WT and cKO animal weights are not significantly different from one another. Error bars, SEM.

D-I Impact of loss of DGCR8 or DICER on localization of the kinase ATR as well as subsequent targets. Pachytene-staged spermatocytes with XY chromosomes (white arrows) from wild-type control (D,G), *Dgcr8* cKO (E,H) and *Dicer* cKO (F,I) mice, stained with anti-SYCP3 (green), anti-ATR (D-F, in red, source: GeneTex GTX70133) and anti-ATR/ATM Substrate (G-I, in red, source: Cell Signaling #5851).

J-K Defective spermatocytes able to progress beyond the pachytene checkpoint. *Dgcr8* cKO diplotene-staged spermatocyte in which both X and Y-chromosomes have circularized with themselves with clear γH2AX localization restricted to the typical sex body boundaries (J). *Dicer* cKO diplotene-staged spermatocyte with circularization of Y and fusion of X to an autosome in which silencing mark γH2AX has spread to entire structure (K).

L Sex chromosome abnormalities do not lead to a global failure to silence key sex chromosome genes *Zfy1* and *Zfy2*. The relative abundance of the *Zfy1* and *Zfy2* transcripts were quantified in purified pachytene spermatocytes from sibling mice of wild-type and *Dgcr8* or *Dicer* cKO genotypes. qPCR assays were performed using cDNA made as described in the Materials and Methods section. The assay was performed with TaqMan probes (Applied Biosystems, Carlsbad CA, USA; Zfy1: Mm00494343_g1 Lot- 1061650, Zfy2: Mm00494350_m1 Lot-965381, ActB: 4352341E - 1102015) and run on a Lightcycler 480 (Roche). Error bars indicate the 95% confidence interval. Significantly different fold change values ($P < 0.01$) are marked with an asterisk.

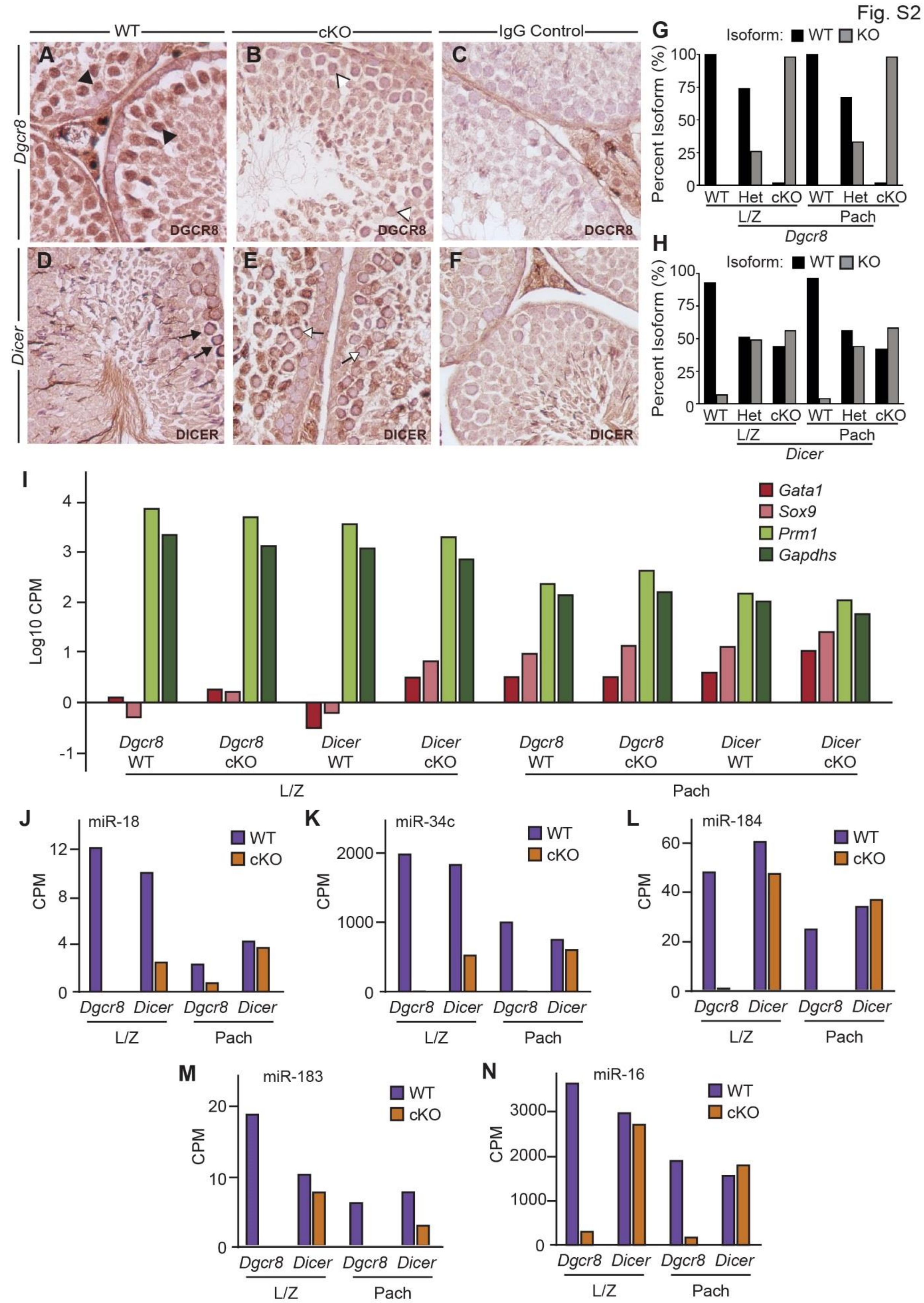

Fig. S2
WT
cKO
IgG Control
Dgcr8
Dicer
A
B
C
D
E
F
DGCR8
DICER
G
Isoform: WT KO
Percent Isoform (%)
WT Het cKO WT Het cKO
L/Z
Pach
Dgcr8
H
Isoform: WT KO
Percent Isoform (%)
WT Het cKO WT Het cKO
L/Z
Pach
Dicer
I
Log10 CPM
Gata1
Sox9
Prm1
Gapdhs
Dgcr8 WT
Dgcr8 cKO
Dicer WT
Dicer cKO
L/Z
Pach
J
miR-18
K
miR-34c
L
miR-184
M
miR-183
N
miR-16
WT
cKO
CPM
Dgcr8 Dicer Dgcr8 Dicer
L/Z
Pach

Fig. S2. Assessment of gene knockdown efficacy and spermatocyte purification from *Dgcr8* and *Dicer* cKO males.

A-F Immunohistochemical localization of DGCR8 (A-C) protein on testis sections from wild-type (WT; A) and cKO (B) mice, together with a negative control IgG-stained WT section (C). Immunohistochemical localization of DICER (D-F) protein on testis sections from WT (D) and cKO (E) mice, together with a negative control IgG-stained WT slide (F). Rabbit anti-DGCR8 antibody was obtained from Proteintech (10996-1) and rabbit anti-DICER antibody was obtained from Novus Biological (NBP1-71691). DICER localizes to the cytoplasm of spermatogonia and spermatocytes in WT testes (D, black arrows), while DGCR8 localization is strongest within the nucleus of primary spermatocytes (A, black arrowheads). Importantly, while DGCR8 signal is completely absent in cKO spermatocytes (B, white arrowheads), DICER signal persists in the cytoplasm of cKO males, albeit at reduced intensity (E, white arrows). Positive signal corresponds to strong brown staining, background signal intensity can be judged from the IgG controls.

G-H The relative abundance of the WT and knockout transcript isoforms of *Dgcr8* (G) and *Dicer* (H) were quantified in purified cells from two different stages of spermatogenesis, leptotene/zygotene (L/Z) and pachytene (Pach), from sibling mice of each genotype (WT: wild type; HET: heterozygote; and cKO). The knockout isoform of *Dgcr8* was not detected in WT *Dgcr8* samples; conversely the WT isoform of *Dgcr8* was detected at only very low levels in *Dgcr8* cKO samples. While the knockout isoform of *Dicer* was apparently detected at very low levels in WT *Dicer* samples, this is likely an artifact due to the nature of the qPCR assay; however the WT isoform of Dicer constituted about 50% of the *Dicer* transcript detected in both *Dicer* cKO samples. qPCR assays were performed as described for ATM in the Materials and Methods section, and used the following primers: Dgcr8_wtExon3 (TGGAGAGACAAGTGTACAGCC and AGGCAATGGCTCTGTAGGTG); Dgcr8_ΔExon3 (TTTCTCCTATGAGGTCGTGGC and GATCCATCCATCAGGCAATGG); Dicer_wtExon22/23 (TGGCTTCCTCCTGGTTATGTG and GTTTGCCATTAGCCAGCAAGC); Dicer_ΔExon22/23 (CTGTTTTGCACGTACCCTGATG and TTGGGGACTTCGATATCCTCTTC); GAPDH (TGAAGCAGGCATCTGAGGG and CGAAGGTGGAAGAGTGGGAG).

I RNA-Seq was used to quantify the transcript levels (CPM, Counts Per Million, as $\log_{10}$(CPM) values) of genes previously reported to show cell-type specific expression in the testis (*Shima et al., 2004, Biol. Reprod. 71, 319–30*) in purified leptotene/zygotene (L/Z) and pachytene (Pach) spermatocytes from *Dgcr8* or *Dicer* WT or cKO mice. *Prm1* and *Gapdhs* were used as markers of meiotic germ cells, and *Gata1* and *Sox9* were used as markers of potentially contaminating somatic cells, specifically Sertoli cells. Leptotene/zygotene purified cells show the lowest levels of somatic markers, with the largest difference appearing between the *Dicer* WT and cKO samples. Pachytene samples show slightly higher levels of somatic markers, with the *Dicer* cKO sample once again having the highest expression of somatic markers.

J-N Small RNA-Seq was used to quantify the mature microRNA transcripts from miRNA hairpins in purified leptotene/zygotene (L/Z) and pachytene (Pach) spermatocytes from *Dgcr8* or *Dicer* WT or cKO mice. Shown here are mature miRNA CPMs for three miRNAs with preferential expression in the germ line: miR-18 (J), miR-34c, the most highly expressed germline miRNA (K), and miR-184 (L). In all cases, germline-specific miRNAs are virtually absent in *Dgcr8* cKOs in all populations of purified cells, while *Dicer* cKOs show a strong reduction of most germline-preferential miRNAs in leptotenze/zygotene purified cells, but not in the pachytene purified cells. Taken together, the results (E, H, I-L) suggest that the *Dicer* cKO samples contain elevated somatic contamination, as compared to either WT controls or the *Dgcr8* cKO samples. Also shown are miRNA CPMs for miR-183 (M) and miR-16 (N), two miRNAs that, in addition to miR-18, regulate ATM.

Fig. S3

A

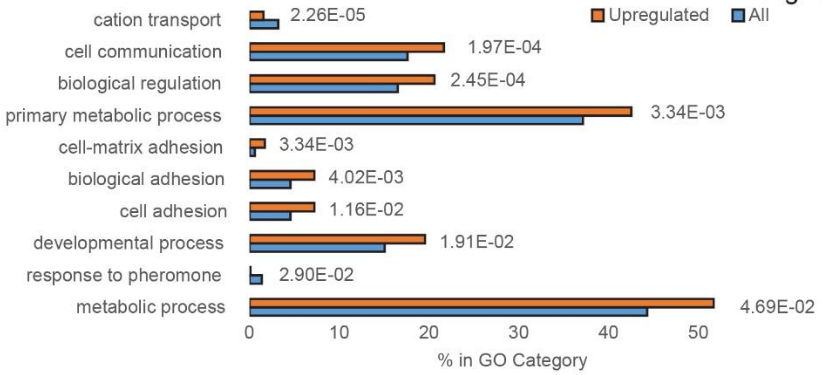

B

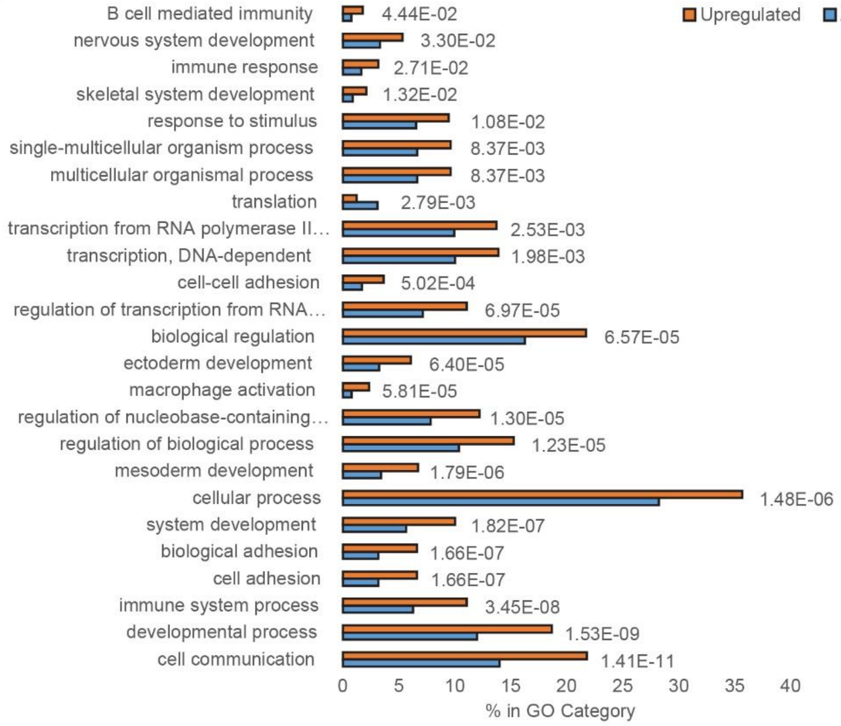

C

Pathw ay	Fold Enrichment	p-value
Apoptosis signaling pathw ay	4.05	5.33E-07
Integrin signalling pathw ay	3.19	6.88E-06
Gonadotropin releasing hormone receptor pathw ay	2.29	1.50E-03
Toll receptor signaling pathw ay	3.61	1.15E-02
EGF receptor signaling pathw ay	2.48	4.49E-02

D

Pathw ay	Fold Enrichment	p-value
Integrin signalling pathw ay	2.52	8.30E-05
Gonadotropin releasing hormone receptor pathw ay	2.24	8.16E-04
Angiogenesis	2.46	2.05E-03

Fig. S3. Gene ontology and pathway analysis of genes upregulated in both *Dgcr8* and *Dicer* cKOs.

A-D RNA-Seq was used to quantify transcript levels in purified leptotene/zygotene (L/Z) and pachytene (Pach) spermatocytes from *Dgcr8* or *Dicer* WT or cKO mice. We identified those genes which were upregulated 1.5x or more in both the *Dgcr8* and *Dicer* cKO, as compared to the wild-type. We then used PANTHER (*Thomas et al., 2003, Genome Res., 13: 2129-2141*) to identify biological processes (A, B) and pathways (C, D) in which these upregulated genes are overrepresented as compared to all expressed genes at leptotene/zygotene (A, C) and pachytene (B, D).

Fig. S4

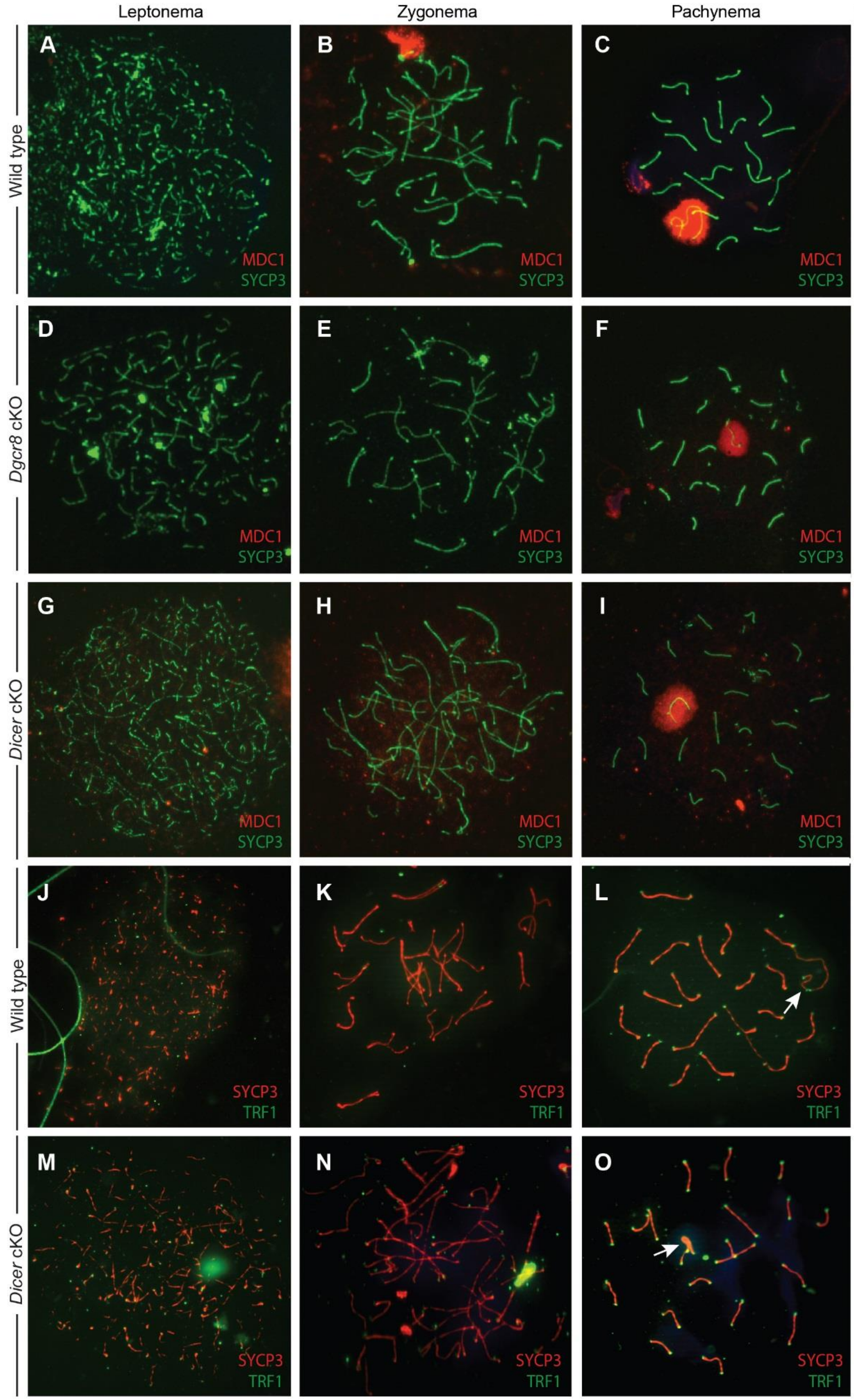
Leptonema
Zygonema
Pachynema
Wild type
Dgcr8 cKO
Dicer cKO
Wild type
Dicer cKO
A
B
C
D
E
F
G
H
I
J
K
L
M
N
O
MDC1
SYCP3
SYCP3
TRF1

Fig. S4. MDC1 and TRF localization in prophase I chromosome spreads from *Dicer* and *Dgcr8* cKO male mice.

A-I Spermatocytes from wild-type (A-C), *Dgcr8* cKO (D-F), and *Dicer* cKO (G-I) mice were stained with anti-SYCP3 (green) and anti-MDC1 (red; Raimundo Freire, Tenerife, Spain) antibodies. Localization of MDC1 protein does not appear to be significantly changed between WT and the *Dicer* cKO or *Dgcr8* cKO throughout prophase I.

J-O Spermatocyte nuclei from the leptotene, zygotene, and pachytene stages of prophase I from wild-type (A-C) and *Dicer* cKO (D-F) mice stained with anti-SYCP3 (red) and anti-TRF1 (green, Abcam ab10579) antibodies. Localization of TRF1 protein does not appear to change between wild type and the *Dicer* cKO throughout prophase I.

Table S1

Gene	*Transcript ID*	*Dgcr8 L/Z log2FC*	*Dicer L/Z log2FC*	*Dgcr8 Pach log2FC*	*Dicer Pach log2FC*
ATM	NM_007499	0.87*	1.19*	0.91*	1.33*
ATR	NM_019864	0.52	0.21	-0.26	0.31
RAD51	NM_011234	0.27	1.00*	-0.50	-0.02
MDC1	NM_001010833	0.01	0.20	-0.22	0.06
RNF8	NM_021419	0.39	0.56	-0.30	0.15
CDK2	NM_183417	-0.98	2.03	-0.06	-0.31
SPO11	NM_001083959	-1.84	-2.43	1.48	1.05
DMC1	NM_010059	1.01	0.73	0.42	0.60
PRDM9	NM_144809	0.66	0.73	0.86	2.24*
MRE11	NM_018736	0.13	-0.09	0.26	-0.18
CHK1	NM_007691	0.13	-0.23	0.47	0.60
CHK2	NM_016681	-0.40	1.53	0.93	1.16
NBN	NM_013752	0.17	-0.38	-0.12	0.25
MLH1	NM_026810	0.30	0.59	0.07	0.16
MLH3	NM_175337	0.48	-0.06	-0.12	-0.06
HORMAD1	NM_026489	-0.10	0.43	-0.07	0.72
HORMAD2	NM_029458	-0.11	0.51	0.23	-0.19

Table S2

Gene	*Transcript ID*	*Dgcr8 L/Z log2FC*	*Dicer L/Z log2FC*	*Dgcr8 Pach log2FC*	*Dicer Pach log2FC*
TPP1	NM_009906	1.28	8.14*	0.27	1.96*
POT1a	NM_133931	0.65	0.84	-0.03	0.07
TINF2	NM_145705	-0.50	-0.05	-0.66	-0.29
TERF1	NM_009352	-0.07	-1.09	0.34	-0.26
TERF2	NM_009353	0.26	0.10	0.18	0.11

Table S3

Gene	*Transcript ID*	*Dgcr8 L/Z WT*	*Dgcr8 L/Z cKO*	*Dicer L/Z WT*	*Dicer L/Z cKO*	*Dgcr8 Pach WT*	*Dgcr8 Pach cKO*	*Dicer Pach WT*	*Dicer Pach cKO*
ATM	NM_007499	6.383	11.703	11.969	27.319	4.269	8.050	5.286	13.330

Table S4

Gene	*Transcript ID*	*Dgcr8 L/Z log2FC*	*Dicer L/Z log2FC*	*Dgcr8 Pach log2FC*	*Dicer Pach log2FC*
C/EBPb	NM_009883	0.75	2.63*	-0.11	1.55*
GADD45a	NM_007836	2.19	3.65*	0.73	2.61*
MCL1	NM_008562	-0.03	0.32	0.09	0.26
PPARG	NM_001127330	not expressed	-1.08	not expressed	-2.88
TPT1 (p21)	NM_009429	-0.56	-0.09	0.09	0.10
TP53	NM_001127233	2.81	3.64	1.58	2.58

Supplementary Tables 1-4. Quantification of transcripts for genes involved in DNA damage repair and telomere protection. The transcriptome was sequenced from purified leptotene/zygotene (L/Z) and pachytene (Pach) cells isolated from either *Dgcr8* or *Dicer* cKO mice, as well as from wild-type littermates as a control. Cells were derived from a minimum of 2 mice per genotype. Each individual cKO sample was compared to a paired wild-type littermate using the transcriptome sequencing analysis package edgeR to determine differential expression.

Table S1 The log_2 of the fold change (cKO CPM/wild-type CPM) for genes involved in double-strand break formation and DNA damage repair, including for *Atm*. For Tables S1, S2, and S4, log_2 fold change values representing more than a 1.5-fold upregulation appear red, and those representing more than a 1.5-fold downregulation appear blue. Significantly different log_2 fold change values ($P < 0.05$) are marked with an asterisk.

Table S2 The log_2 of the fold change (cKO CPM/wild-type CPM) for genes encoding shelterin proteins, which function to protect the telomeres.

Table S3 Individual CPM values determined by transcriptome sequencing for *Atm*.

Table S4 The log_2 of the fold change (cKO CPM/wild-type CPM) for genes transcriptionally regulated by ATM (*Jang et al., 2010, Experimental & Molecular Medicine 42, 195-204; Takagi et al., 2015, Cell Reports 10, 957–967; Bruno et al., 2006, Cancer Cell. Dec;10(6):473-86*)
